# Synthesis and Studies of Electro-Deposited Yttrium Arsenic Selenide Nanofilms for Opto-Electronic Applications

**DOI:** 10.3390/nano10081557

**Published:** 2020-08-08

**Authors:** Chawki Awada, Goodfriend M. Whyte, Peter O. Offor, Favour U. Whyte, Mohammed Benali Kanoun, Souraya Goumri-Said, Adil Alshoaibi, Azubike B. C. Ekwealor, Malik Maaza, Fabian I. Ezema

**Affiliations:** 1 Department of Physics, College of Science, King Faisal University, P.O. Box 400, Al-Ahsa 31982, Saudi Arabia; cawada@kfu.edu.sa (C.A.); mkanoun@kfu.edu.sa (M.B.K.); 2 Nano Research Laboratory, Department of Physics and Astronomy, University of Nigeria, Nsukka 410001, Nigeria; goodfriend.whyte@unn.edu.ng (G.M.W.); azubike.ekwealor@unn.edu.ng (A.B.C.E.); 3 Metallurgicaland Materials Engineering Department, University Nigeria, Nsukka 410001, Nigeria; peter.offor@unn.edu.ng (P.O.O.); favour.otung@unn.edu.ng (F.U.W.); 4 College of Science, Department of Physics, Alfaisal University, P.O. Box 50927, Riyadh 11533, Saudi Arabia; sosaid@alfaisal.edu; 5 Nanosciences African Network (NANOAFNET), iThemba LABS-National Research Foundation, 1 Old Faure Road, P.O. Box 722, Somerset West 7129, Western Cape Province, South Africa; maaza@tlabs.ac.za or; 6 UNESCO-UNISA Africa Chair in Nanosciences/Nanotechnology, College of Graduate Studies, University of South Africa (UNISA), P.O. Box 392, Muckleneuk Ridge, Pretoria 0001, South Africa

**Keywords:** nanocomposite, rare-earth ion, binary chalcogenide, electro-deposition, density functional theory

## Abstract

Nanocomposite films grown by incorporating varying concentrations of Yttrium, a d-block rare-earth ion, into the binary chalcogenide Arsenic selenide host matrix is here presented. Films were grown via the wet-chemical electro-deposition technique and characterized for structural, optical, surface morphology, and photoluminescence (PL) properties. The X-ray Diffraction (XRD) result of the host matrix (pristine film) showed films of monoclinic structure with an average grain size of 36.2 nm. The composite films, on the other hand, had both cubic YAs and tetragonal YSe structures with average size within 36.5–46.8 nm. The fairly homogeneous nano-sized films are shown by the Scanning Electron Microscopy (SEM) micrographs while the two phases of the composite films present in the XRD patterns were confirmed by the Raman shifts due to the cleavage of the As-Se host matrix and formation of new structural units. The refractive index peaked at 2.63 within 350–600 nm. The bandgap energy lies in the range of 3.84–3.95 eV with a slight decrease with increasing Y addition; while the PL spectra depict emission bands across the Vis-NIR spectral regions. Theoretically, the density functional theory (DFT) simulations provided insight into the changes induced in the structure, bonding, and electronic properties. Besides reducing the bandgap of the As_2_Se_3_, the yttrium addition has induced a lone pair *p*-states of Se contributing nearby to Fermi energy level. The optical constants, and structural and electronic features of the films obtained present suitable features of film for IR applications as well as in optoelectronics.

## 1. Introduction

Pragmatic research on nanocomposite films for integrated photonics (IP) and optoelectronic applications is on the rise [1,2]. Synthesizing and fabricating these nanocomposites for IPs require nanomaterials with characteristics non-linear optical features, as noted by Bardosova et al. [3]. Binary and ternary amorphous chalcogenide materials such as As_2_S_3_, As_2_Se_3_, and Ge_2_Sb_2_Te_5_ are already gaining ground in research due to their high optical nonlinearity, high refractive index, structural stability, and NIR/MIR transparency [4,5,6]. Besides, the innate quality of low phonon energy of these metal chalcogenides makes them a rich host for incorporating rare-earth ions with a significant effect on their optical properties [7,8]. However, little or no detailed literary report is found for these materials’ crystalline phase since they possess phase-change property dependent on some propaedeutic conditions and with wide applications in optical waveguides [9], infrared lasers [10], narrow bandgap semiconductors for photovoltaic infrared imaging [11], double-shell quantum dots fluorescent probe for bio-labeling and bio-imaging of cancer cells [12], downconverting layers in silicon solar cells [13], and phase-change memory (PCM) device for data storage [14], among others.

With a focus on PCM devices, much greater speed and lower power drain during data storage, processing, and information dissemination are attained when circuits are fabricated with chalcogenide-based materials [4]. Several techniques have been employed to synthesize these materials; they include RF magnetron sputtering [15], melt-quenching [16], pulsed laser deposition (PLD) [17], thermal evaporation [18], spin-coating [19], among others. 

In this work, the wet-chemical electro-deposition (ED) technique was used to synthesize a Yttrium-doped arsenic selenide film dispersed on a fluorine-doped SnO_2_(FTO) glass substrate under room temperature. This technique offers a better alternative to the afore-listed techniques in terms of reduced cost, deposition time, less complicatedness during use, size control, and reproducibility of films [20]. Interestingly, the ED technique has also been successfully deployed to synthesize crystalline chalcogenide-based PCM films, window layer material for solar cells, and optoelectronic applications [20].

## 2. Experimental Details

The major analytical grade chemicals employed in this survey are Yttrium (III) nitrate hexahydrate (Y(NO_3_)_3_·6H_2_O, 99.8%); Selenium (Se, 99.98% purity) metal, and Arsenic trioxide (As_2_O_3_, 99.98%); they are the sources of Y^3+^, Se^2−^ and As^3+^ ion, respectively. Other chemicals include acetone, distilled water, hydrochloric acid, and ethanol. All the reagents were procured from Sigma-Aldrich Chemie, Munich, Germany except for the distilled water prepared in the laboratory.

For the preparation of the pristine film (Y0%), 2.5 mL of 0.3 M HCl (Sigma-Aldrich Chemie, Munich, Germany) was used to dissolve 0.1 M As_2_O_3_ and 0.1 M Se in a 100 mL beaker separately. Distilled water was added to each of the containers and continuously stirred for 10 min to enhance the uniform dissolution of the reacting species. The separate solutions were mixed in the volume ratio of 1:1 in a 50 mL beaker and deposited on a pre-cleaned Fluorine-doped Tin Oxide (FTO) glass substrate of sheet resistance, 23 Ω/sq. The grown films on the glass substrates served as the working electrodes in a three-electrode potentiostatic cell supplying a 10 V dc voltage for 10 s.

Similarly, the preparation of the composite films involved mixing separate solutions of 0.1 M As_2_O_3_, 0.1 M Se, and 0.05 M Y(NO_3_)_3_·6H_2_Oin a simple volume ratio of 2:2:1 in a 50 mL beaker. The dopant was varied in a molar concentration of 0–4 mol% and deposited via a similar process as for the pristine film. To enhance purity, crystallinity, and eliminate absorbing oxides, films were annealed at a temperature of 200 °C. 

The structural, optical, and morphological features of the films were examined from X-ray diffraction and Raman spectroscopy; UV-Vis-NIR and photoluminescence (PL) spectroscopy; and Scanning Electron Microscopy (SEM), respectively. The XRD patterns were recorded using a Bruker D2 phaser table-top model diffractometer (Bruker, Madison, WI, USA) in the 2θ scanning range of 15–80°, while the Raman spectra was collected using a confocal Raman microscope (LabRAM HR800, Horiba Scientific, Villeneuve-D’Ascq, France) in a backscattering geometry with a spectral resolution of 0.3 cm^−1^ at ambient temperature. A He–Ne laser of λ = 633 nm and a power level of 2 mW was employed. The lower power was chosen in order to prevent any photo-induced effect [21]. The same microscope was used to obtain the PL data at an excitation wavelength of 325 nm. The UV-1800series Shimadzu spectrophotometer(Schimadzu Schweiz GmBH Inc., Reinach, Suisse) in the wavelength interval of 200–1100 nm was used to obtain the optical data while the Jeol, JSM 7000 series Scanning Electron Microscope (Jeol Ltd., Tokyo, Japan) was used to obtain the morphological micrographs of films at 15.0 kV scan voltage.

## 3. Results and Discussion

### 3.1. XRD and Raman Analysis

**Figure 1:**
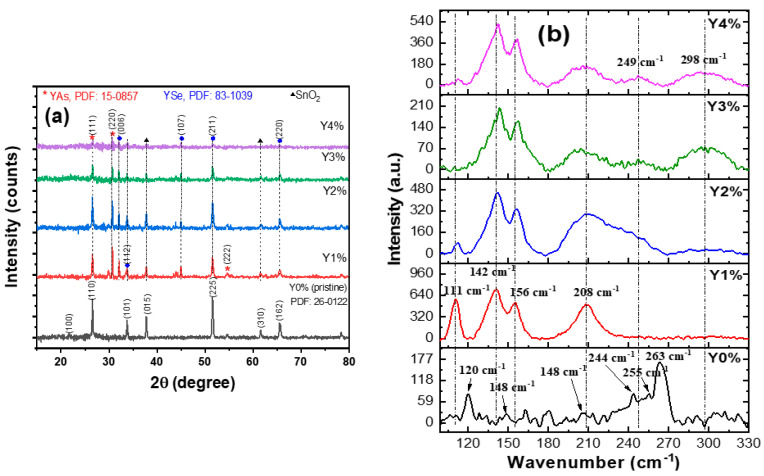
(**a**) XRD pattern (**b**) Raman spectra of pristine and composite films.

The XRD patterns depicted in Figure 1a were used to analyze the films’ structure.

The diffraction patterns as seen in Figure 1a revealed polycrystalline nanocomposite films with peaks at 2θ values of 26.55°, 37.75°, and 51.52° for (110), (015), and (225) orientation planes, respectively, for the pristine film. On the other hand, peaks at 2θ = 26.60°, 30.70°, 54.72°, and 2θ = 32.14°, 33.79°, and 51.39° corresponding to crystal planes of (111), (220), (222) and (006), (112), and (211), respectively, were observed for the composite films. Other reflection peaks present could be attributed to having originated from the substrate material.

The peaks in the diffractograms for the pristine film showed a good match with the Powder Diffraction File (PDF) no.: 26-0122 [22], revealing a monoclinic crystal structure, while the composite films exhibited cubic Yttrium arsenide (YAs) and Tetragonal Yttrium selenide (YSe) phases with PDF card nos.: 15-0857and 83-1039, respectively. The occurrence of the two phases was in a mixed form such that no particular composition had a unique phase.

The Debye-Scherrer relation: $D=\frac{K\lambda }{\beta \mathrm{Cos}\theta }$ [23] was used to calculate the crystallite sizes. Estimation of other crystal variables such as the lattice strain (ε=β4tanθ), inter-planar distance (d=λ2sinθ), and dislocation density ($\delta =\frac{I}{D^{2}}$) were done and reported in Table 1.

From Table 1, the pristine film gave a size of 34.5 nm, while nano-sized films in the range of 38.98–46.86 nm were estimated for the composite films. The increase in the average crystallite size of the composite film is most likely attributed to the microstrain, although in minute amounts, due to the presence of the Y^3+^ ions in the system [24]. However, the strain (ε) analysis presents positive values as seen in Table 1, which vividly suggests the presence of a biaxial tensile strain in the composite films during nucleation [25]. The relatively large strain values of 1% and 4% films are also reflected in the surface morphologies, as shown in the SEM micrographs.

On the other hand, Figure 1b reveals Raman phonon modes for the pristine and Y/As_2_Se_3_ composite film. Raman spectrum of pristine shows different phonon modes located at 120 cm^−1^, 148 cm^−1^, 206 cm^−1^, 244 cm^−1^, 255 cm^−1^, and 263 cm^−1^. The peaks of 148 cm^−1^ and 255 cm^−1^ are attributed to the vibration of covalent bonds in Se_8_ rings and the bending modes in Se units [26,27]; the peak of 206 cm^−1^ is attributed to As_4_Se_4_ cages [28] and the peaks of 244 cm^−1^ and 263 cm^−1^ are attributed to As-Se-Se-As dimers [29]. However, the peak located at 120 cm^−1^ is not identified; it could also be attributed to structural units containing As-As bonds [28]. 

After introducing 1% of Yttrium, most of the peaks observed in pristine disappear and new phonon modes attributed to new structures appear. This substantial change in Raman spectra confirms the formation of new structural units consisting of both As and Se atoms.The three peaks of 244 cm^−1^, 255 cm^−1^, and 263 cm^−1^ disappear; however, a new high peak is observed at 208 cm^−1^. This peak is related to the molecules As_4_Se_4_ cages, observed at 206 cm^−1^ in pristine, and the shift is expected when the composition is highly altered. This can be explained by the breaking of Se bonds in the Se_8_ rings and As-Se-Se-As dimers and in turn, the formation of more As-rich networks with the formation of monomeric As_4_Se_4_ cages based on realgar–type structure [29]. However, this peak decreases and becomes broader, after introducing 2% of Y. If we look more profoundly at the spectrum of 2%, we can see a peak around 249 cm^−1^ overlapping with the peak of 208 cm^−1^; in addition, another weak peak starts to appear around 298 cm^−1^. After introducing of 3% of Y, the two new peaks at 249 cm^−1^ and 298 cm^−1^ become more pronounced. By adding 4% of Y, the three mentioned peaks become broader and more intense. This change explains the increasing of disorder of Se-Se chains and consequently the increase of the presence of the amorphous phase in the matrix [30]. 

Another three peaks also appear when we introduce Yttrium. The first two peaks are located at 142 cm^−1^ and 156 cm^−1^, and these are close to the peak located at 148 cm^−1^ of Se_8_ ring. The two peaks could be attributed to AsSe_3_ pyramidal units. AsSe_3_ is formed after the breaking of As-As or Se-Se bonds in As and Se rich units. The transition between the two phases can be explained by the blue shift when the Yttrium concentration varies between 1% and 3%, then a redshift is observed when the Y concentration varies between 3% and 4%. The latter is due to the deterioration effect resulting from the high incorporation of Yttrium in the matrix and this is also accompanied by the appearance of the amorphous phase as explained above. In their quantitative study of the dependence of composition on the peak position of AsSe3, Yanget al. showed that the peak of AsSe3 modes shifts toward lower energy when As content increases from 0 till 0.4 and it shifts toward higher energies when As content increases from 0.4 to 0.6 [31]. The third peak is located at 111 cm^−1^; this peak is sharper than others and it decreases by increasing the Y content. It could be attributed to the As_4_Se_4_ or As_4_Se_3_ cages because it presents the same behaviour of the peaks located at 208 cm^−1^. The sharpness of this peak reflects the crystallinity existing in each Y/As_2_Se_3_ composite and it correlates as well with the XRD patterns, which, in turn, showed less crystallinity in the nanocomposites with a high Y concentration. The predominance of the amorphousness phase observed in 4% is consistent with the high number of strains and dislocation for the 4% presented in Table 1 of XRD patterns.

### 3.2. Optical Studies

#### 3.2.1. Transmittance (*T*), Absorption Coefficient (*α*), Energy Gap (*E_g_*), and PL Plots

**Figure 2:**
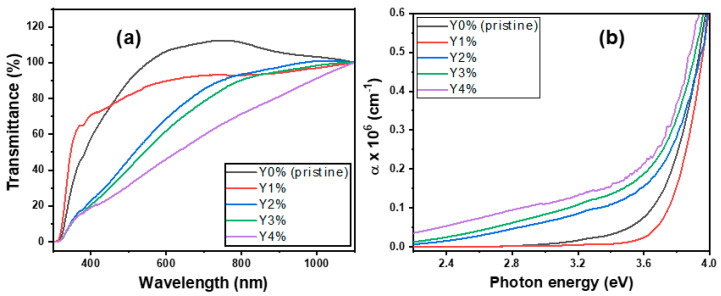
(**a**) Transmittance and (**b**) absorption coefficient plots of pristine and composite films.

**Figure 3:**
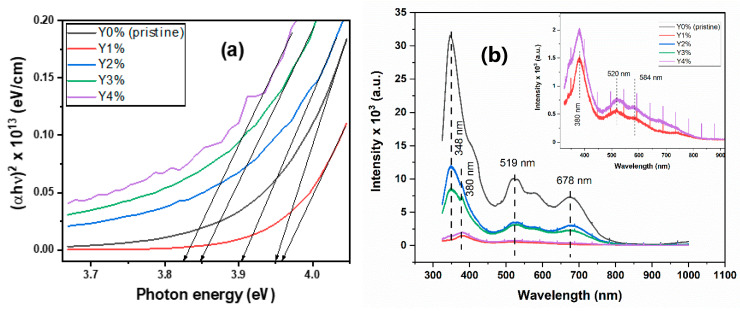
(**a**) Energy bandgap and (**b**) PL spectra of pristine and composite.

The transmittance and absorption coefficient plots are shown in Figure 2a,b while the energy bandgap (*E_g_*) and photoluminescence (PL) plots are depicted in Figure 3a,b respectively.

The pristine and composite film’s transmittance is depicted in Figure 2a. The optical relation: $T=10^{-A}$ was employed for its calculation, where *A* is the film’s absorbance. Generally, the film’s transmittance increased with wavelengths but decreased with concentration. An increase in concentration gradually led to an increase in film thickness with fewer incident photons passing through the material; hence, a consequent drop in transmittance [32]. Nevertheless, transmittance values over 90% were recorded for films within wavelengths of 500–1000 nm. Highly transmitting selenide-based metal chalcogenide films in the optical and IR windows coupled with their respective wide energy gap makes for good application in selective coatings for solar cells according to the literature [33].

Employing the transmittance (*T*) and reflectance (*R*) data, the absorption coefficient was determined from: $\alpha =\frac{1}{t}\ln\left(\frac{{\left(1-R\right)}^{2}}{T}\right)$ where *t* = film thickness [25]. The film thickness (*t*) was calculated using the gravimetric weight difference technique ($d=\frac{M}{A\times \rho }$) where *M*, *A*, and ρ represents the mass, area, and density of film, respectively [34]. The thickness values are given as: Y0% = 130.0 nm; Y1% = 117.0 nm; Y2% = 131.2 nm; Y3% = 139.4; Y4% =145.2 nm.

The plot of α against the photon energy (*hν*) shows vividly an increase with photon energy and a decrease with concentration. The film’s absorption coefficient is in the order of 10^6^ cm^−1^. However, at the absorption edge (3.6 eV), 1% composition showed the least α value of 0.02 × 10^6^ cm^−1^ while 4% had the peak absorption of 0.21 × 10^6^ cm^−1^. The significant increase in α-values with radiation energy and concentration affords the nanocomposites good suitability for optoelectronic applications [35].

On the other hand, the Taucrelation (αhυ)1n=A(hυ−Eg) was employed to estimate the film’s energy gap (*E_g_*); all parameters retaining their usual meaning with *n* = ½ for direct allowed transition [36]. The energy gap values for the pristine and composite films were derived from the projection of a straight line onto the energy (*hυ*) axis, where α is zero as depicted in Figure 3a. The film’s energy gap was lowered with Yttrium concentration, except for 1% concentration (3.96 eV), which had the highest *E_g_* value. This may be due to its relatively large crystal defects, as shown in the SEM micrograph. However, values in the interval of 3.82–3.96 eV were recorded for the films. More so, theoretical investigations reveal that the energy gap lowering was due to the strong hybridization between Y-4d and Se-4p. Nevertheless, lowering of energy gap due to Yttrium incorporation enhances the movement of free electrons to the conduction band from the valence band [37].

The relationship between an absorbed photon of energy and the consequent photoemissions was studied from the photoluminescence (PL) spectra for the pristine and composite films, as presented in Figure 3b.

**Figure 4:**
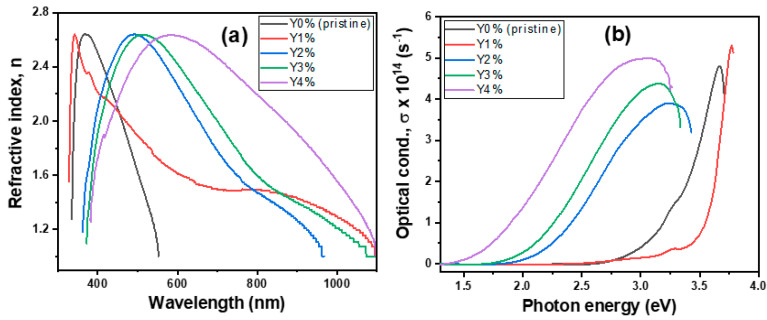
(**a**) Refractive index and (**b**) optical conductivity plots of pristine and composite films.

The wavelength of the excitation source was set near the material’s absorption wavelength of 325 nm. From Figure 3b, it is observed that the pristine film had strong PL emission peaks at 348 nm and 380 nm, which represent a near-band-edge (NBE) transition. This is subsequently followed by the film at 2%, 3%, 4%, and 1% concentration in decreasing order of intensities. The very low intensities of emission bands observed at Y1% and Y4% are primarily due to high crystal defect (dislocation), as revealed in SEM micrographs (see Figure 4b), and also the relatively high biaxial strain factor, as seen in the diffraction patterns. Additionally, the sharp spikes or lines found in 1% and 4% correspond to emissions due to crystal defects, suggesting that multi-photon absorption was involved from these defect centers [38].

However, the additional broad emission peak at 678 nm for the deep-red light, although with low intensity, is characteristic of Yttrium-based semiconductors used in manufacturing fluorescent lamps. This peak is characteristic of chalcogenide films with broad bands and peaks near midway of the energy gap (*E_g_*/2 = 1.90 eV [λ = 652 nm]). This behavior is attributed to defects recombination with a consequent large Stokes shift [39,40].

Nanocrystals are usually associated with two types of emissions, namely: band-edge (excitonic) and trapped (delayed) emissions. The categorization depends largely on the proximity of the emission peaks to the material’s absorption edge [41]. Hence, the luminescence spectra for both the pristine and composite films at λ_interval_ = 500–700 nm in Figure 3b suggests a trapped-type of emission due to broad and Stokes-shifted peaks. In contrast, however, excitonic emission peaks are usually sharp and positioned near the film’s absorption edge [42].

#### 3.2.2. Refractive Index (n) and Optical Conductivity (σ)

Figure 4a,b depicts the plots of *n* versus λ as well as σ versus *hν* (energy) for varying percentage molar concentrations of the pristine and composite films. The index of refraction was calculated using: $n=\frac{1+R}{1-R}+\sqrt{\frac{4R}{{\left(1-R\right)}^{2}}-K^{2}}$, where all variables retain their usual meaning [43].

The pristine and composite films recorded a high refractive index value of 2.63, irrespective of wavelength. However, 2%, 3%, and 4% concentrations reveal a slight shift and spectral broadening in the visible region, which subsequently dropped to a value of 1.00 in the near-infrared region. The high *n* value for the pristine film is relatively close to the reported value of 2.83 [44]. A fairly close result of 2.7 was also obtained for a solution-phase grown As_2_Se_3_ film annealed at 150 °C [45]. Nevertheless, the non-linear refractive index of films simply reveals its non-dependence on the wavelength on a linear scale. This is one interesting property for chalcogenide-based materials for PCM applications.

The optical conductivity was calculated using $\sigma =\frac{\alpha nc}{4\pi }$ and depicted in Figure 4b. Both the pristine and composite films reveal high optical conductivities in the order of 10^14^ s^−1^. However, at the energy level near the mid-range of the bandgap (1.9 eV), 2%, 3%, and 4% concentrations showed relatively higher conductivities (~10^14^ s^−1^), as opposed to the pristine and 1% film with lower values (~10^12^ s^−1^). Hence, the higher the σ values, the higher the tendency for photon absorption and electronic excitation. The low σ value of the 1% film is also reflected in the low emission peaks observed in the PL spectra. Comparatively, it is discovered that the σ values of Erbium-doped As-Se films in an earlier report possess relatively higher values, which enhance optical conductance by well over 10^2^ times than yttrium-doping [46].

#### 3.2.3. Complex Dielectric Plots

**Figure 5:**
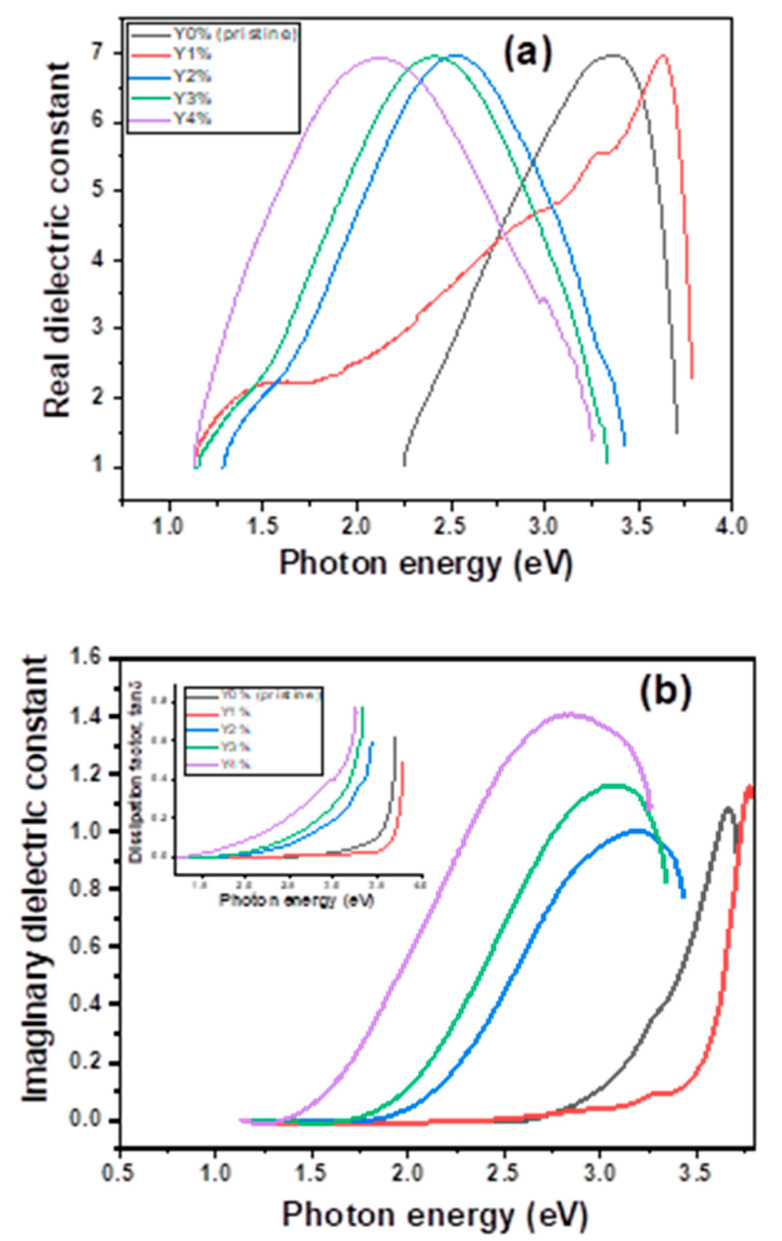
(**a**) Real dielectric (**b**) Imaginary dielectric plots of pristine and composite films.

Figure 5a,b shows the real and imaginary dielectric plots of the films. The complex dielectric function was estimated from the relation ε = ε_r_ + iε_i_ [47], where ε_r_ = n^2^ − k^2^ is the real dielectric constant and ε_i_ = 2nk is the imaginary dielectric constant.

The thin films have values of real dielectric constants of 7.03 between photon energies of 2 and 3.6 eV. The real dielectric constant is a measure of retardation of the speed of light through a given material. The higher the value, the more the speed of light propagation is retarded in the material of interest. A material with a high real dielectric constant would readily find application as an effective coating. The imaginary dielectric constant, on the other hand, is a measure of how much a dielectric material absorbs and stores energy in an electric field as a result of dipole motion [47]. In an actual sense, this is a measure of charge absorption and storage by a given dielectric material. Its value is given as 1.4 at 2.8 eV for the Y4% concentration, as shown in Figure 5b. A material with a high value of imaginary dielectric constant will readily find application as a capacitor used for charge storage. Unfortunately, the nanocomposites thus grown lack good charge storage capacity due to relatively low values of the imaginary dielectric constant.

In addition, the radiation dissipation factor (loss factor) given by the ratio of the imaginary to the real dielectric i.e., Tan δ = ε_i_/ε, was also estimated. This is shown in the inset in Figure 5b as a function of photon energy (eV). It is seen that the dissipation factor of the material increased with increasing photon energy and concentration.

### 3.3. Surface Analysis

**Figure 6:**
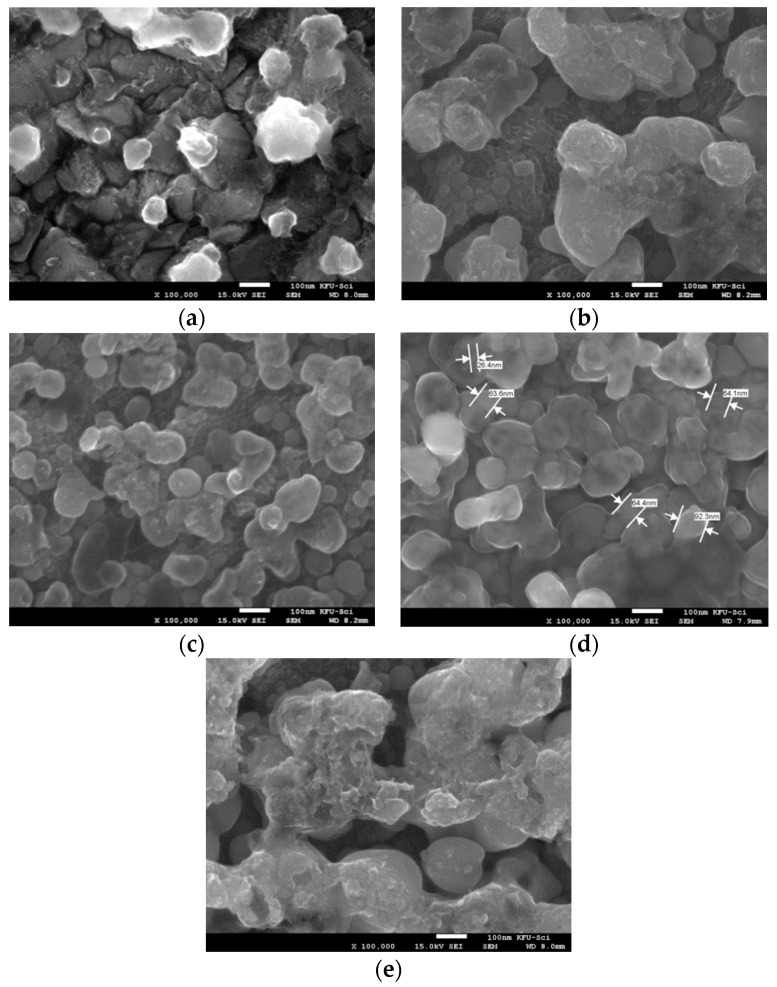
SEM micrographs of (**a**) Pristine film (Y0%) (**b**) Y1% (c) Y2% (**d**) Y3% (**e**) Y4% films.

The micrographs revealing the surface features of the pristine and composite films are depicted in Figure 6a–e:

The SEM micrographs in Figure 6 depict films with average diameters (sizes) as follows: Y0%: 50.02, Y1%: 43.66, Y2%: 42.16, Y3%: 45.10, and Y4%: 47.93. These mean particle sizes are in fairly good trend with the XRD data reported in Table 1. In addition, from Figure 6, films at Y2% and Y3% revealed fairly smooth, spherical, uniform, and homogeneous nano-balls with relatively less crystal defects, while Y1% and Y4% films revealed large, rough, and agglomerated particles with grain boundaries and cracks. These defects reflect the presence of relatively large strain in the 1% & 4% system, probably arising during growth or heat treatment, which also in essence could most probably be the epicenter of the sharp emission spikes in the PL spectra. Furthermore, the conspicuously large grain boundary and dislocation density observed in Y4% had a significant effect on the crystallinity of the composite films, which is also consistent with the observed low peak intensities in the XRD pattern (see Figure 1) [19]. The phenomenon of near-loss of crystallinity in Y4% results when the strain-induced stacking fault intersecting the surface particles introduces additional dynamical beams, which interfere to produce a band of fringes near the fault. A dislocation emerging at the surface perturbs the local lattice orientation and therefore changes (reduces) the reflected beam intensities, often leading to destructive interference (zero beam intensity) [48].

### 3.4. Electronic Structures

**Figure 7:**
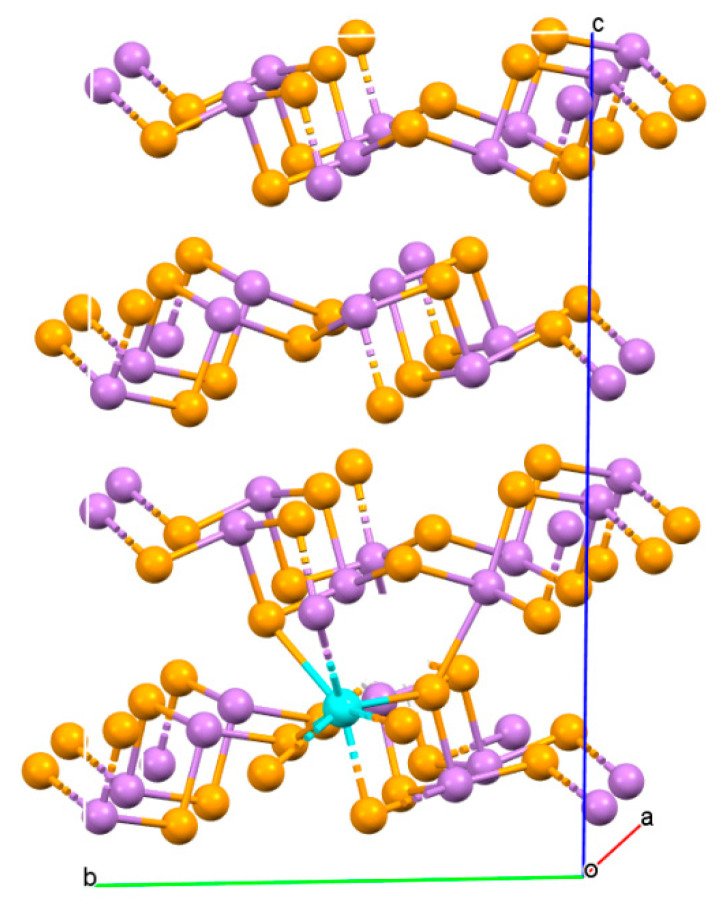
Crystal structure of Y doped As_2_Se_3_ along the crystal axes, as is shown in violet; Se in orange and Y in cyan.

To investigate the impact of the incorporation of Y in As_2_Se_3_ regarding its electronic and bonding properties, the DFT computation within the generalized gradient approximation in form of Perdew-Burke-Ernzerhof (PBE) level [49] as implemented in ATK [50] package based on local combination of the atomic orbital method is performed. It is well known that the *GGA* functionals generally underestimate band gaps for semiconductors and other systems. GGA+*U* calculations were also carried out as well to describe the d states of the Y atom. Here, U (equal to what is often called U_eff_ = U − J = 6 eV) is used as the on-site interaction term based on the “around mean-field” (AMF) implementation, as proposed in refs [51,52,53,54]. The string Brillouin zone k-point was adjusted to be 10 × 10 × 10, and the periodic wave function was described using 150 Ry cutoff energy. The As_2_Se_3_ structure was relaxed at the experimental lattice parameters. The optimized lattice parameters of *a* = 4.502 Å, *b* = 10.845 Å, *c* = 12.998 Å show a fair agreement with the experimentally reported values [55] of *a* = 4.505 Å, *b* = 10.967 Å, *c* = 12.915 Å within average deviations less than 1.0%. The Y/As_2_Se_3_ system was modeled with a supercell of 2 × 2 × 1 corresponding to the doping level of 3.125%, approaching the experimental concentration. In the optimized geometry, it can be observed that Yttrium atom connects six selenium atoms and one arsenic atom to form YSe_6_As-cluster, as illustrated in Figure 7. Moreover, the average bond lengths lie in the range of 2.844–3.211 Å and 3.342 Å for Y−Se and Y−As bonds, respectively.

**Figure 8:**
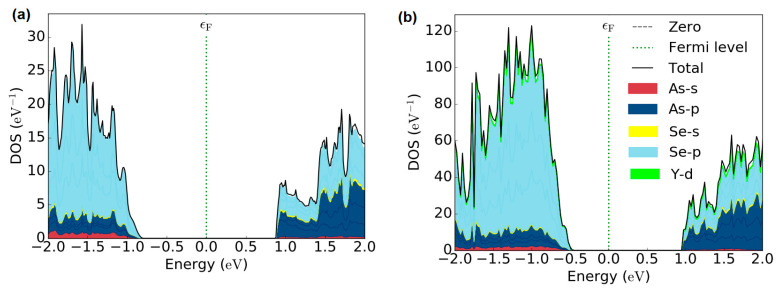
Calculated total and partial density of state of (**a**) As_2_Se_3_ and (**b**) Y doped As_2_Se_3_system. The vertical dashed line denotes the Fermi level.

The electronic structures of pure As_2_Se_3_ and as well as Y doping are shown in Figure 8, where the site projected density of states (PDOS) plots on individual atoms are presented. As seen in Figure 8a,b, the valence band of the parent arsenic selenide has dominant contributions from 4p states of the Se, with additional contributions from the 4p states of the As atoms and s states of the Se and As atoms. In the case of Y addition, there is a strong hybridization between Y-4d and Se-4p states near the top of the valence band. This hybridization might seem to be the origin of the decrease in the bandgap as Y addition increases. For the conduction band, the 4p states of the Se and Y 4-d states largely contribute to the parent system and Y/As_2_Se_3_ system, respectively. We can conclude that the optical measured and computed band gap values for the samples indicate a decrease upon Y doping in pure As_2_Se_2_.

**Figure 9:**
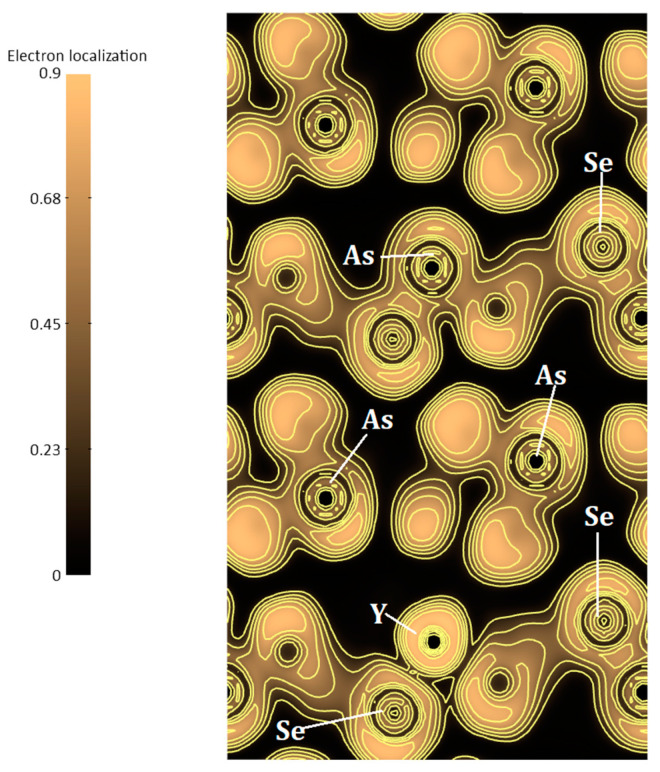
Electron-localization function (e/Å^3^) in (100) plane of Y addition in As_2_Se_3._

We calculated the electronic localized function (ELF) [56] with the aim to understand the bonding character changes induced by Yttrium doping in As_2_Se_3_. ELF is a dimensionless localization index bounded within (0, 1) and constitutes a relevant parameter to comprehend the bonding in As_2_Se_3_. We will obtain perfect electron localization in bonding when ELF = 1.We display in Figure 9, the ELF maps of yttrium doped As_2_Se_3_. The high ELF values show the existence of covalent bonds, inert cores, or lone pairs. Definitely, the interaction between the rare earth element (Y) and neighboring atoms is mainly ionic in the present yttrium doped As_2_Se_3_. It is also vividly seen that almost zero ELF values (≈0.20) exist for Y and Se atoms. These values are almost insignificant for assigning strong covalent bonds, but can certainly be attributed to Se 4p with As 4p states hybridization. The bonding character analysis is fitting observations made on Raman spectra. Furthermore, when we examined the arrangement in the crystalline structures, we could see Se atoms occupying different layers; thus, they interact differently with Y atoms. It is to be mentioned that in the same behavior was reported in the pure As_2_Se_3_ [49], where a strong interaction produced lone-pair states, which lie farthest away from the midpoint of the lone-pair band.

## 4. Conclusions

In this work, nanocomposite films of Y/As_2_Se_3_ were successfully grown via the solution-phase electro-deposition technique. The incorporation of rare-earth ions into the host material with the subsequent formation of two mixed phases of composites was successfully carried out. This is vividly seen from the presence of new structural units due to the varying concentrations of Yttrium ions, as shown in the Raman spectra. More so, crystal defects such as grain boundaries due to strain during nucleation had a profound effect on the structural, morphological, and optical performance of the films. This is especially revealed in the diffraction patterns, surface micrographs, and photoluminescence emissions at certain molar concentrations. Besides, high optical transmittances across the Vis-NIR window, lowering of the bandgap energy with increasing Yttrium content, and high red-shifted non-linear refractive index of pristine and composite films are reported.

Nevertheless, to gain some theoretical insight into the yttrium doping, we further performed first-principles investigations using density functional theory. The calculations of the electronic properties of Y doped As_2_Se_3_confirm the reduction of the energy bandgap compared to the pure As_2_Se_3_material. We also gained insights into the bonding characterization changes due to yttrium doping. These qualities afford our material much suitability for potential applications in optoelectronics as well as phase-change memory devices and solar cell accessories.

## Figures and Tables

**Table 1:** Summary of crystal variables for pristine and composite films.

Sample	2θ (°)	d (Å)	FWHM (β/rad)	(hkl)	D_av_ (nm)	δ (line/m^2^) × 10^15^	ε × 10^−3^
**Y0%(pristine)**	26.55	3.3534	0.0036	110	40.04 ± 0.009	0.62 ± 0.003	3.76 ± 0.007
	37.78	2.3793	0.0039	015	37.65 ± 0.002	0.70 ± 0.005	2.84 ± 0.003
	51.57	1.7708	0.0050	225	31.01 ± 0.005	1.03 ± 0.009	2.56 ± 0.009
**Average values**					**36.23** **± 0.005**	**0.78 ± 0.006**	**3.05 ± 0.006**
**Y1%**	26.56	3.3533	0.0041	111	34.12 ± 0.005	0.85 ± 0.008	3.42 ± 0.001
	30.70	2.9099	0.0025	220	55.51 ± 0.003	1.16 ± 0.007	3.58 ± 0.008
	51.58	1.7708	0.0056	211	27.32 ± 0.008	1.33 ± 0.008	2.91 ± 0.005
**Average values**					**38.98 ± 0.007**	**1.11 ± 0.007**	**3.30 ± 0.004**
**Y2%**	26.53	3.3570	0.0043	111	33.15 ± 0.007	0.90 ± 0.009	2.55 ± 0.006
	30.63	2.9164	0.0029	220	48.72 ± 0.006	0.42 ± 0.001	1.69 ± 0.003
	51.56	1.7711	0.0057	211	27.70 ± 0.007	1.30 ± 0.001	1.98 ± 0.004
**Average values**					**36.52 ± 0.006**	**0.87 ± 0.003**	**2.07 ± 0.001**
**Y3%**	26.56	3.3533	0.0039	111	36.34 ± 0.009	0.75 ± 0.006	2.15 ± 0.001
	30.70	2.9099	0.0019	220	74.70 ± 0.009	0.17 ± 0.009	1.75 ± 0.002
	51.57	1.7708	0.0056	211	27.27 ± 0.006	1.34 ± 0.004	2.92 ± 0.001
**Average values**					**46.10 ± 0.008**	**0.75 ± 0.006**	**2.27 ± 0.001**
**Y4%**	26.57	3.3496	0.0021	111	69.04 ± 0.008	0.20 ± 0.009	3.18 ± 0.003
	30.71	2.9090	0.0027	220	52.78 ± 0.006	0.35 ± 0.008	3.47 ± 0.009
	51.58	1.7705	0.0082	211	18.78 ± 0.003	2.83 ± 0.004	4.24 ± 0.001
**Average values**					**46.86 ± 0.006**	**1.12 ± 0.007**	**3.93 ± 0.004**
